# Assessment of Language Barriers Between Dental Students and Patients in Riyadh, Saudi Arabia—A Mixed Methods Study

**DOI:** 10.3390/dj14020115

**Published:** 2026-02-14

**Authors:** Sanjeev B. Khanagar, Samar Alanazi, Razan Alotaibi, Hebah Alenazi, Lujain Altalhi

**Affiliations:** 1 Preventive Dental Science Department, College of Dentistry, King Saud bin Abdulaziz University for Health Sciences, Riyadh 11426, Saudi Arabia; 2 King Abdullah International Medical Research Centre, Riyadh 11481, Saudi Arabia; 3 Ministry of the National Guard Health Affairs, Riyadh 11426, Saudi Arabia; 4 College of Dentistry, King Saud bin Abdulaziz University for Health Sciences, Riyadh 11426, Saudi Arabia

**Keywords:** barriers, communication, dental, focus groups, language, misinterpretation, patients, terminologies, understanding

## Abstract

**Background**: Language serves as a significant barrier to accessing dental services. Dental treatment options are often complex and involve terminology that is unfamiliar to most patients. In some cases, dental students may use technical terms that patients do not understand, leading to confusion and misunderstandings. Therefore, this study aimed to assess the language barriers faced by dental students and patients in Riyadh, Saudi Arabia. **Methods**: A mixed-methods research design was employed to evaluate language barriers between dental students and patients, as it provides an in-depth understanding and generates information beyond mere numerical data. The study was conducted from 1 September 2024, to 30 August 2025, in Riyadh, Saudi Arabia. Data collection primarily involved conducting interviews with focus group members using a comprehensive topic guide consisting of predetermined questions. **Results**: Forty dental students and forty patients agreed to participate in this study. The students encountered significant difficulty explaining terms such as crown lengthening (72.5%) and periodontitis (67.5%), while patients reported limited understanding of interim removable dental prosthesis (65%) and fixed dental prosthesis (60%). Comparative analysis indicated that sixth-year students reported significantly more difficulty explaining “crown lengthening” and “prefabricated post and core” compared to fifth-year students. It was also observed that patients’ educational level had a significant impact on their understanding of terms such as “interim dental prosthesis” and “removable dental prosthesis.” Qualitative analysis revealed patients’ partial understanding or misinterpretation of dental terminologies. **Conclusions**: Our findings indicate that language discordance, even among speakers of the same native language, can hinder effective communication, particularly when technical vocabulary is involved. Students may struggle to explain procedures in a manner that patients can easily understand. This can lead to incomplete patient comprehension and potential non-compliance with treatment recommendations. Hence, we recommend incorporating Arabic dental terminologies alongside English into the curriculum, developing bilingual glossaries, and using visual aids when communicating with patients.

## 1. Introduction

Oral diseases are among the most common health problems worldwide. Although largely preventable, they often go untreated, significantly reducing the quality of life for those affected [1]. One contributing factor to this issue may be the underutilization of dental services for preventive care, which can be attributed to poor communication between patients and dentists. Language serves as a significant barrier to accessing dental services [2]. Language barriers also hinder effective communication between healthcare providers and patients, resulting in suboptimal care and dissatisfaction with the services provided. These barriers disrupt treatment adherence and the utilization of preventive and screening services, which further delay the access to timely care, leading to inadequate management of chronic diseases, and ultimately contributing to unfavorable health outcomes [3]. Dental treatment options are complex and involve terminology that is often unfamiliar to most patients. In some cases, dental students may use dental terms that patients do not understand, leading to confusion and misunderstandings [4]. Dentists often assume that patients understand this terminology and frequently overestimate their patients’ literacy levels, increasing the likelihood of misunderstandings [5].

In dentistry, dental practitioners must possess not only strong knowledge, clinical skills, and problem-solving abilities but also competence in effective communication with their patients [6]. Using terminology that is often unfamiliar to laypeople can negatively affect decision-making, treatment adherence, and overall outcomes [7]. Moreover, studies have shown that patients’ limited understanding of dental terminology used by their dentists can significantly impact clinical results [8]. Consequently, information must be conveyed carefully to ensure patient understanding. However, even well-presented information can be misunderstood if patients are unfamiliar with common medical terminology, which may have different meanings for them.

Medical research conducted in Saudi Arabia has highlighted the issue of language barriers affecting communication between healthcare professionals and patients [9]. One study indicated that these language barriers pose significant challenges to medication safety practices [10]. Additionally, research by Mahrous et al. revealed that 42% of patients expressed dissatisfaction with the clarity and language used by healthcare providers when conveying information [11]. Furthermore, a study by Al-Khashan et al. identified the use of medical and technical terminology as a primary barrier frequently encountered in communication [12]. Similarly, research by Wahabi and Alziedan identified language as a barrier to effective communication between patients and healthcare providers, which can lead to non-compliance with treatment instructions [13].

Research conducted in Saudi Arabia indicates that the population has limited health literacy. One study found that approximately 50% of participants exhibited limited health literacy, resulting in insufficient comprehension of medical information [14]. It was recommended that health materials be provided in Arabic to overcome language barriers. Additionally, another study revealed that 59.5% of participants had a low level of health literacy, with language identified as a significant factor affecting patients’ understanding of medication instructions [15].

Effective communication between patients and practitioners has been linked to improved clinical outcomes and increased patient satisfaction [16,17]. However, research indicates that dental students often lack adequate communication skills, highlighting the need for improvement. Several patients have reported a perceived deficiency in empathy and insufficient connection during their treatment [18]. In Saudi Arabia, there are numerous cultures and several languages are spoken. However, Arabic is the official and most widely spoken language. The limited Arabic vocabulary related to dental terminology creates a communication barrier for dental students when interacting with patients, particularly with terms such as recession, periodontitis, gingivitis, post-and-core, and pit and fissure sealant. Despite this challenge, there is limited data on the communication difficulties faced by dental students and their ability to effectively convey dental terminology to ensure patient understanding. Therefore, this study aimed to assess language barriers between dental students and patients in Riyadh, Saudi Arabia. The objectives were to identify the language barriers and difficulties dental students face when communicating with patients, assess patients’ understanding of commonly misunderstood dental terminology during these interactions, and evaluate patients’ comprehension of the procedures explained by dental students.

## 2. Materials and Methods

### 2.1. Research Design

A mixed-method research design was employed to evaluate language barriers between dental students and patients. The study was conducted from 1 September 2024, to 30 August 2025, in Riyadh, Saudi Arabia. Prior to data collection, ethical approval was obtained from the Institutional Review Board at the King Abdullah International Medical Research Center in Riyadh, Saudi Arabia (KAIMRC) (IRB approval No. 0000028224, Study No. NRR24/085/7).

### 2.2. Sample Size Estimation

Based on a random pilot sample of 10 students and 10 patients. The participants’ understanding of the questions was assessed using a dichotomous scale and estimated; therefore, a point-biserial correlation was used to determine the sample size. An effect size of 0.5 indicated a required sample size of 38 in each group, with a 95% confidence interval and 80% study power. The sample size was estimated using G*Power software (version 3.1.9.4; Düsseldorf, Germany).

### 2.3. Sampling Technique

A non-probability convenience sampling method was employed to recruit dental students and patients who attended dental clinics at the College of Dentistry, King Saud bin Abdulaziz University for Health Sciences (KSAU-HS), in this study.

### 2.4. Eligibility Criteria

Inclusion criteria: Patients attending dental clinics at KSAU-HS in Riyadh who were undergoing comprehensive dental treatment and those who could comprehend and converse only in the Arabic language; dental students in their clinical years (5th and 6th years); and participants who have provided written informed consent. Exclusion Criteria: Residents who are not citizens or those younger than 18 years of age. Patients who were not citizens, or those younger than 18 years of age and individuals with cognitive or communication impairments that would prevent participation, were excluded.

### 2.5. Data Collection Tool

A comprehensive topic guide consisting of predetermined questions was developed and refined through collaboration between the principal investigator and all members of the research team. This guide included questions designed to elicit detailed information from participants and was used to ensure consistency across all participants while allowing flexibility to explore emerging concepts. These questions were developed after observing the frequent challenges students encountered with dental terminology when communicating with their Arabic-speaking patients. The guide assessed participants’ demographic details, communication barriers and understanding of common dental terminology (e.g., periodontitis, gingival recession, crown lengthening, pit and fissure sealant, Glass Ionomer Cement (GIC) sandwich technique). The questionnaire was initially developed in English and subsequently translated into Arabic. A bilingual expert conducted the translation, while an independent translator performed a back-translation into English. Collaborative discussions were held between the translators and the researchers to reach an agreement on the translations. The back-translated version was then compared with the original English questionnaire to verify consistency during the translation process. A pilot study involving a random sample of 10 students and 10 patients was conducted to evaluate face validity. Based on the feedback received, no modifications to the assessment tool were deemed necessary.

### 2.6. Data Collection

**Figure 1:**
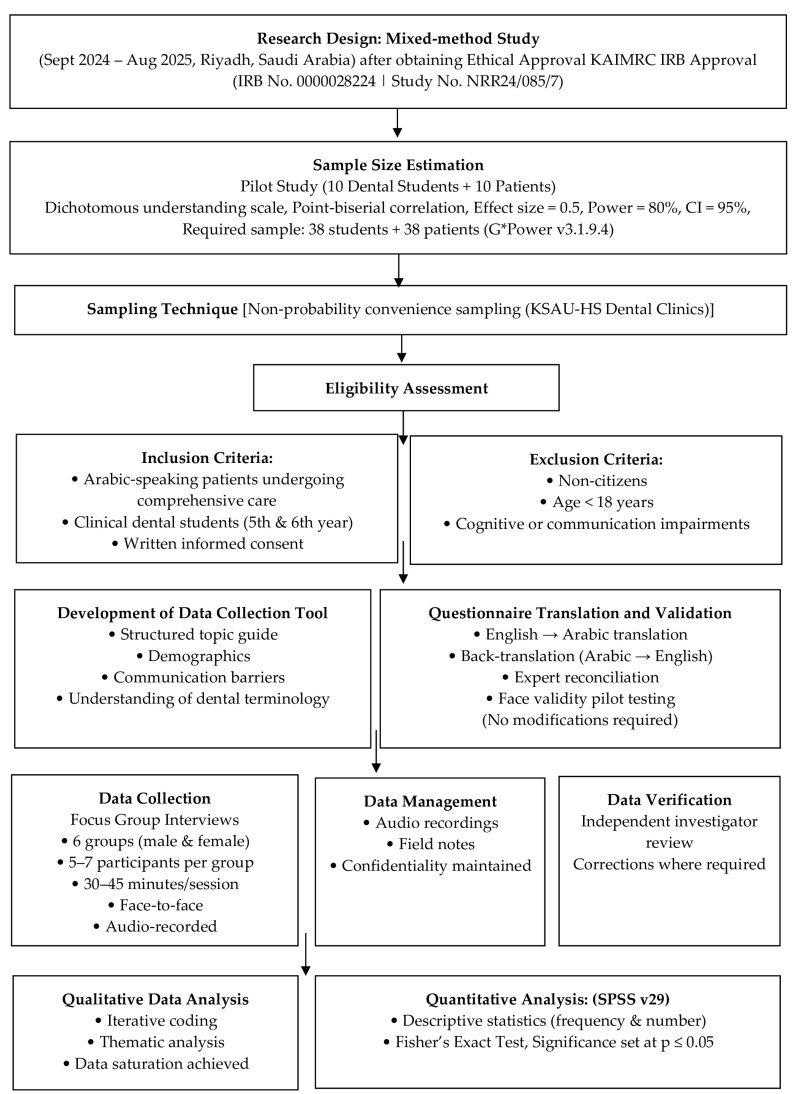
Methodology flowchart.

The data collection method primarily involved conducting interviews with six focus groups, separated by gender into male and female groups, each consisting of 5 to 7 members representative of participant population. Participants were assured that their anonymity and confidentiality would be maintained. The interviews lasted between 30 and 45 min and were mainly conducted face-to-face in quiet patient waiting rooms without any disturbances. This was facilitated by scheduling patients and students from the same clinical session together. Prior to the start of each interview, the study objectives were explained. For each focus group, the purpose of the discussion was explained, and the investigator obtained consent to audiotape the session. Audiotaping was used to preserve the integrity of the data. The procedures were explained to the participants, who then shared their experiences and opinions in response to questions based on the interview guide. One of the investigators, who was trained, was responsible for asking the questions and guiding the discussion, while the other two investigators listened to the interview and took notes in addition to the audiotaping. The research team engaged in reflexive practices throughout the study to acknowledge and minimize potential researcher bias. The focus group discussions were facilitated by trained dental interns who were fluent in Arabic and familiar with the sociocultural contexts of both patients and dental students. While this insider position facilitated rapport and in-depth discussion, the researchers remained aware of their professional roles and the potential influence on participants’ responses. Reflexive notes were maintained after each focus group to document assumptions, impressions, and emerging interpretations, which were revisited during data analysis. An independent fourth investigator, unaware of the initial assessment, reviewed the audio recordings to verify whether the information gathered from the participants had been accurately recorded by the other investigators. Corrections were made wherever necessary. Data saturation was established through iterative analysis, conducted to code the data until the codes reached a point of stability and no additional codes appeared [Figure 1—Methodology Flowchart].

Multiple procedures were employed to examine the collected data. Initially, the audio recordings were transcribed and cross-checked against the handwritten field notes to ensure accuracy and completeness. The transcripts from each focus group were then saved as word processing files. Each researcher reviewed the recordings and read through the transcripts multiple times to further verify the data’s reliability. The analysis was conducted in two phases.

Content analysis involved counting the frequency of specific words or concepts appearing in the transcripts. This method corresponds to the manifest level, providing a straightforward description of the data [19,20,21,22].

This study employed Thematic analysis, which is a qualitative research method used to identify, analyze, and interpret recurring patterns or “themes” within data sets such as interview transcripts or open-ended survey responses. The process involves thoroughly familiarizing oneself with the data, coding various segments, and developing themes to capture the underlying meanings, typically related to the research questions. This flexible approach allows researchers to gain a deeper understanding of individuals’ opinions, experiences, and perspectives [23,24,25].

The analysis began with initial familiarization with the data, initial coding, and identifying various themes and ideas that emerged from the transcripts. Audio recordings were transcribed verbatim and independently reviewed by multiple members of the research team to ensure accuracy. Initial open coding was conducted line by line to identify meaningful units of data. The principal investigator, with good research experience, undertook the coding and identification of themes based on inductive thematic analysis. This process comprises familiarization with the data, formulating initial codes, identifying the themes, reviewing themes, defining and naming themes, and writing the report. These codes were then compared, discussed, and refined through team consensus, resulting in the development of higher-order categories and themes. Once the themes were developed, consensus was reached among the co-investigators. Discrepancies in coding were resolved through discussion, and themes were continuously refined until conceptual clarity and internal coherence were achieved. The data were then examined to locate content that could be categorized under these themes. Subsequently, themes and emerging concepts were recoded to create more clearly defined categories [23]. To ensure the reliability of these qualitative findings, individual co-investigators independently analyzed the data and compared their interpretations.

### 2.7. Statistical Analysis

The data analysis was conducted using SPSS software, Version 29 (IBM Corporation, Armonk, NY, USA), and descriptive statistics were calculated and presented in number and frequencies. Fisher’s Exact Test was applied to assess the association between demographic details and participants’ responses. Statistical significance was set at *p* ≤ 0.05. The data from the focus group discussions were thematically analyzed by the researchers.

## 3. Results

In the present study, 60 dental students and 100 patients were approached; however, only 40 students and 40 patients, respectively, expressed their willingness to participate.

This study employed collecting quantitative and qualitative data concurrently from dental students and patients within the same clinical setting. The quantitative component assessed the prevalence and distribution of communication barriers and the understanding of dental terminology, while the qualitative component explored patients’ experiences, perceptions, and contextual explanations of these barriers. Both datasets were analyzed separately and then integrated during interpretation to provide a comprehensive understanding of language barriers in dental settings.

### 3.1. Demographic Details of the Study Participants

The study participants were predominantly female, comprising 24 (60%) of the dental students and 27 (67.5%) of the patients. Arabic was the first and native language for 38 (95%) of the dental students and 39 (97.5%) of the patients, respectively. Sixth-year students were primarily involved in the study, accounting for 21 students (52.5%). Approximately 26 (65%) of the patients held a diploma or bachelor’s degree. Among the patients, 15 (37.5%) were in the 18–25 age group. Table 1 and Table 2.

### 3.2. Self-Reported Comprehension of the Study Participants Towards Questions Related to Dental Terminologies

When dental students were asked about the difficulties they face in describing various dental procedures to their patients, the majority reported challenges with explaining crown lengthening (29, 72.5%), followed by periodontitis (27, 67.5%). Other commonly difficult terms included prefabricated post and core (18, 45%), inlay/onlay restoration (17, 42.5%), indirect pulp capping (17, 42.5%), and internal bleaching (17, 42.5%). Fewer students reported difficulty explaining plaque-induced gingivitis (1, 2.5%), pit and fissure sealants (4, 10%), fixed dental prosthesis (6, 15%) and re-root canal treatment (8, 20%).

Most patients reported difficulty understanding interim removable dental prosthesis (26, 65%), followed by periodontitis (25, 62.5%), fixed dental prosthesis (24, 60%), the GIC sandwich technique (23, 57.5%), pit and fissure sealants (23, 57.5%), removable dental prosthesis (22, 55%), and gingival recession (21, 52.5%). Fewer than 25% of patients had difficulty understanding inlay/onlay restorations (7, 17.5%), indirect pulp capping (5, 12.5%), and crown lengthening (4, 10%). Table 3.

### 3.3. Association Between Demographic Variables and Patients with Their Responses

The analysis indicated no statistically significant differences between males and females in their comprehension of any dental terminology. It was noted that in terms of age-related differences, the term “crown lengthening” was the sole term that exhibited statistical significance (*p* = 0.012), with only patients aged 26–35 reporting an understanding of this term. In relation to educational level differences, two dental terms, “interim dental prosthesis” (*p* = 0.002) and “removable dental prosthesis” (*p* = 0.032), demonstrated statistical significance. Table 4.

### 3.4. Association Between Demographic Variables of Students with Their Responses

The analysis indicated that 6th-year students (90.5%) reported significantly more difficulty explaining “crown lengthening” compared to 5th-year students (52.6%) (*p* = 0.007). Additionally, 6th-year students (66.7%) reported much greater difficulty explaining “Prefabricated Post and Core” compared to 5th-year students (21.1%) (*p* = 0.004). All 6th-year students (100%) reported no difficulty explaining “fissure sealants,” whereas 21.1% of 5th-year students reported difficulty (*p* = 0.027). Regarding gender-related differences, 25% of female students reported difficulty explaining “fixed dental prostheses,” while no male students (0%) reported any difficulty. Table 5.

### 3.5. Thematic Analysis of the Patient Responses

All patients who participated were asked to describe their understanding during communication with their dentist regarding their diagnosis and the procedures employed during treatment. Fifteen predefined themes were included in the questions, with approximately 2 to 4 codes emerging from each theme. The major themes that patients found difficult to understand are discussed below:

*Interim Removable Dental Prosthesis:*

Most patients who indicated that they understood the meaning of an interim removable dental prosthesis were aware that it is a temporary replacement. One 24-year-old female patient explained, “These are temporary prosthetics that are artificial teeth temporarily placed to replace missing or treated teeth, until permanent prosthetics are installed”. “After tooth extraction to compensate for the space, an alternative to implants’ (Response by a 31-year-old male patient).

*Periodontitis:*

Patients perceived periodontitis as inflammation of the tissue surrounding the tooth and were aware that it was associated with bleeding. A 39-year-old male responded with “Persistent and difficult to treat inflammation”, while a 24-year-old male responded to is by saying that “It is a long-term condition that affects the gum tissue surrounding the teeth. It occurs because of the buildup of bacteria and plaque (tartar) on the teeth and gums, leading to irritation and inflammation of the gum tissue.” Patients also reported it as the cause of redness, pain, halitosis and sensitivity with the gums.

*Fixed Dental Prosthesis:*

Most patients did not know exactly what a fixed dental prosthesis was; they often considered it to be a device for protecting the tooth, such as a veneer or crown. “It is formulated to preserve teeth”, responded a 33-year-old female.” It is a post root canal treatment formula” responded a 35-year-old male. About 25% of the patients considered it as a fixed replacement. “A fixed prosthesis is an artificial device that is permanent replacement to missing teeth, such as a bridge or crown, and can only be removed by a dentist” was the response by a 27-year-old female.

*GIC Sandwich Technique:*

The GIC sandwich technique is associated with procedures involving temporary restoration, permanent restoration, or non-restorative procedures such as a cleaning procedure of tooth. “Putting the filling between two materials” (43-year-old male), “Procedure related to temporary filling” (38-year-old female), “A technique in which a layer of cement is used as a base under a composite filling to protect the tooth and enhance adhesion” (26-year-old male) were some of the responses.

*Pit and Fissure Sealant:*

Patients described pit and fissure sealants as agents for caries prevention or protection, fracture prevention, restoration, or pulp protection. A 30-year-old female responded with “A plastic layer used mostly for mild tooth decay”. A 37-year-old male responded “Nerve protection”. A 42-year-old female responded “clean the tooth from decay”. A 28-year-old female responded “A procedure that aims to protect molars from decay”.

Other themes and their responses are included in Table 6.

## 4. Discussion

A qualitative approach was employed to explore the language barriers experienced by students and patients. This research design is well-suited for exploratory studies because it does not seek to produce numerical answers to research questions. Instead, it develops a framework that facilitates understanding of phenomena within its natural context, focusing on individuals’ perceptions, experiences, and perspectives. Additionally, it allows participants to express their own priorities, thereby avoiding a common limitation of questionnaire-based research, which often reflects the researcher’s preconceived notions. Another advantage is the use of focus groups, which can be organized by clinical session groups, ideally comprising participants of the same gender but diverse backgrounds. This method provides in-depth information and collects data from multiple people more quickly and at a lower cost than interviewing each participant individually [26].

The results of the current study indicate that the majority participants had visited a dentist within the previous year. We observed that a significant proportion of dental students experience difficulties in effectively communicating specific dental terms, such as crown lengthening, periodontitis, and prefabricated post and core. Similarly, many patients consistently reported confusion or misunderstanding regarding procedures like interim removable dental prosthesis, periodontitis, and fixed dental prosthesis. These findings highlight a critical communication gap in dental practice, despite the shared Arabic language between students and patients. This gap reflects the limited dental vocabulary available in Arabic, which complicates students’ ability to accurately translate or simplify complex dental concepts in a way that patients can easily understand. For example, while “periodontitis” was often explained by students using scientific terminology, patients frequently interpreted it in more general terms such as “gum swelling,” “bleeding,” or “pain,” indicating a partial but incomplete understanding of the condition.

In the present study, a good number of the patients held a diploma or bachelor’s degree. However, the persistence of a high level of misunderstanding of health information suggests that the issue is not solely linguistic but also related to poor health literacy, as reported in previous studies in Saudi Arabia [14,15]. Dental students appeared to underestimate the difficulties associated with terms such as gingival recession, pit and fissure sealant, re-root canal treatment, and fixed and removable dental prostheses. More than half of patients still reported difficulties in understanding these terminologies. This discrepancy reflects potential overconfidence, limited bidirectional communication, and a lack of feedback from patients to verify their comprehension. This issue can be addressed by incorporating feedback tools, such as the “teach-back” method, which assess patient understanding by having them explain the information in their own words to ensure comprehension.

Patients often misunderstand periodontitis, perceiving it merely as bleeding gums. They may also mistakenly believe that crown lengthening involves the removal of gum tissue, and sometimes even bone. These misconceptions can cause fear, affect their willingness to clear doubts and lead to the avoidance of necessary dental care. Psychosocial factors that influence understanding and act as communication barriers in dental care are complex, often arising from patient anxiety, the use of complicated technical language, and limited consultation time. These factors directly affect how patients interpret and respond to clinical information [27]. Such barriers can result in serious issues, including patients misunderstanding treatment plans, failing to provide valid consent, and not adhering to post-operative instructions. The thematic analysis in the current study revealed that patients frequently use everyday analogies and substitute technical terms with their own interpretations. These behaviors serve as psychosocial indicators of gaps in health literacy, coping mechanisms, and efforts to reduce uncertainty. These cues reflect a patient’s emotional, social, or mental state, which can influence their health outcomes and ability to engage in care [28]. Therefore, incorporating approaches such as shared decision-making—a collaborative process in which healthcare providers and patients work together to make informed care choices—empowers patients to select treatment plans that improve their experiences and clinical outcomes [29]. Another important concept is patient activation, where patients take an active role in managing their health, leading to better health outcomes, behaviors, and overall experiences [30].

Research documented in the literature indicates that the lack of culture-specific vocabulary and the presence of stigma can make it challenging for individuals to describe health conditions. Additionally, the absence of language support or culturally appropriate services may hinder timely diagnosis and access to healthcare [31,32]. Encounters within the healthcare system where the patient and provider do not share a common language increase the risk of poor communication, misdiagnosis, medication errors, complications, and even death [33,34]. Studies show that language barriers negatively affect health outcomes, access to care, healthcare utilization and costs, the effectiveness of healthcare providers, as well as patient satisfaction and safety [2,33].

Our findings align with prior research indicating that language discordance, even among speakers of the same native language, can hinder effective communication, particularly when technical vocabulary is involved. Almansour et al. [35] observed that Arabic-speaking medical students often resorted to using English medical terms or vague Arabic equivalents due to the lack of standardized translations for complex medical concepts. This results in incomplete patient understanding and potential non-compliance with treatment recommendations. Furthermore, a study by Almusharraf et al. [36] revealed that both Arabic-speaking and non-Arabic-speaking students encountered challenges in clinical communication due to insufficient training in using context-appropriate Arabic medical language. They proposed incorporating standardized Arabic medical terms into the curriculum to enhance clarity and foster patient trust.

In the present study, although students found pit and fissure sealants and fixed dental prostheses easier to explain, patients still reported high levels of misunderstanding. This discrepancy suggests that students may overestimate their ability to communicate effectively or lack strategies to assess patient comprehension. Such mismatches highlight the need for interactive patient education tools, such as visual aids or simple analogies, to bridge comprehension gaps. The results indicated that sixth-year students reported significantly more difficulty explaining “crown lengthening” and “Prefabricated Post and Core” compared to fifth-year students. This suggests that these procedures are particularly challenging concepts to explain to patients. This difficulty may be due to the complexity of the procedures or the fact that students encounter them more frequently in later clinical years. Additionally, the study observed that patients’ educational level had a significant impact on their understanding of terms such as “interim dental prosthesis” and “removable dental prosthesis”. Diploma/bachelor’s degree holders consistently demonstrated better understanding across most terms, while school graduates demonstrated lower comprehension to most terms.

Consistent with the findings of Almutairi et al. [9], cultural and linguistic diversity among healthcare workers and patients in Saudi Arabia further complicates communication, particularly when technical concepts lack direct Arabic equivalents. A study conducted by Bakhsh et al. [37] highlighted the language and communication challenges faced by emergency department physicians in the Makkah region of Saudi Arabia. The findings revealed that most of the doctors encountered language barriers, while more than half of the emergency department physicians reported that their patients experienced adverse outcomes due to ineffective communication [37]. Another study by Alshammari M. et al. [38] emphasized the language barriers experienced by nurses when communicating with patients in a Saudi Arabian hospital. Seventy-six percent of the nurses reported encountering language barriers frequently or daily, and 48% indicated that this resulted in compromised patient care [38]. A study by Alsharyah HMH and Sayed SAM identified communication obstacles encountered by Saudi nationals when seeking assistance in emergency departments. The primary barrier faced by individuals seeking care in hospitals across Saudi Arabia was healthcare providers who do not speak Arabic [39]. Additionally, a study conducted by Alharazi RM et al. [40] assessed factors and barriers affecting nurses’ communication while providing care to patients in Jeddah, Saudi Arabia. These findings indicated that language barriers between nurses and patients present a significant challenge to effective communication [40]. These findings underscore the importance of translating dental and medical terminologies into Arabic not by entirely replacing English, but by equipping students with parallel Arabic terminology and communication skills relevant to their patient population. A comprehensive strategy is needed, including the development of best practice guidelines for dental care providers, training for interpreters, and implementing policy changes to address the impact of language barriers on healthcare delivery and utilization in Saudi Arabia.

The results of this study will be used to develop Arabic dental terminology into simpler, culturally relevant local terms. This objective can be achieved through collaborative efforts involving dentists, psychologists, and linguistic experts. Subsequently, these terms will undergo a validation process to help enable students to become familiar with Arabic dental terminology and incorporate it into patient history documentation. This initiative can be implemented through workshops. Furthermore, students should be trained in structured communication using simulated patients, which can build their confidence before interacting with real patients. Feedback tools, such as the “teach-back” method, should be adopted to assess patient understanding by having them explain the information in their own words, ensuring comprehension. Additionally, future research will be conducted to evaluate the effectiveness of this approach. The findings can subsequently be submitted to the university’s dental curriculum committee to advocate for incorporating sociolinguistics and culturally competent communication using Arabic terminology alongside English terminology within existing clinical dental courses.

Strengths and Limitations: This research examined the challenges that dental students and patients in Saudi Arabia face when trying to understand common dental terminology. A limitation of the study was its cross-sectional design. However, this approach serves as an independent research method that complements quantitative studies by exploring concepts that are difficult to quantify, thereby clarifying meanings and enhancing comprehension. Another limitation was related to the use of the convenience sampling technique and a single center in this research, which restricts the generalizability of the findings. A comparison was made with studies conducted among medical and nursing professionals, as there were no previous studies conducted in dentistry. Qualitative research is often criticized for its subjectivity and lack of reproducibility; however, these studies help generate hypotheses that can be objectively assessed in future research. These studies are valuable for obtaining important insights from participants and provide a comprehensive understanding of patient values and experiences, both of which are essential for evidence-based practice.

## 5. Conclusions

The findings of the study highlight that dental students face significant challenges in effectively communicating with their patients, particularly when explaining clinical procedures using Arabic terminology. Similarly, patients often interpret these terms in overly general or incorrect ways. Hence, the findings of this study should be considered to develop Arabic dental terminology into simpler, culturally relevant local terms. This goal can be achieved through collaborative efforts involving dentists, psychologists, and linguistic experts.

## Figures and Tables

**Table 1:** Demographic details of the dental students (*n* = 40).

Characteristic	Category	*n* (%)
Gender	Male	16 (40.0)
	Female	24 (60.0)
Age (years)	18–25	40 (100.0)
Primary language	Arabic	38 (95.0)
	English	2 (5.0)
Academic year	5th year	19 (47.5)
	6th year	21 (52.5)

**Table 2:** Demographic details of patients (*n* = 40).

Characteristic	Category	*n* (%)
Gender	Male	13 (32.5)
	Female	27 (67.5)
Age (years)	18–25	15 (37.5)
	26–35	11 (27.5)
	36–45	9 (22.5)
	>45	5 (12.5)
Primary language	Arabic	39 (97.5)
	Other	1 (2.5)
Educational level	Incomplete school education	4 (10.0)
	High school graduate	9 (22.5)
	Diploma/Bachelor’s degree	26 (65.0)
	Postgraduate degree	1 (2.5)

**Table 3:** Self-reported comprehension of the study participants towards dental terminologies.

Dental Term	Dental Students—Experienced Difficulty Explaining *n* (%)		Patients—Understood Meaning *n* (%)	
	Yes	No	Yes	No
Periodontitis	27 (67.5)	13 (32.5)	25 (62.5)	15 (37.5)
Gingival recession	10 (25.0)	30 (75.0)	21 (52.5)	19 (47.5)
Crown lengthening	29 (72.5)	11 (27.5)	4 (10.0)	36 (90.0)
Plaque-induced gingivitis	1 (2.5)	39 (97.5)	9 (22.5)	31 (77.5)
Indirect pulp capping	17 (42.5)	23 (57.5)	5 (12.5)	35 (87.5)
Inlay/Onlay restoration	17 (42.5)	23 (57.5)	7 (17.5)	33 (82.5)
Pit and fissure sealant	4 (10.0)	36 (90.0)	23 (57.5)	17 (42.5)
GIC sandwich technique	13 (32.5)	27 (67.5)	23 (57.5)	17 (42.5)
Internal bleaching	17 (42.5)	23 (57.5)	12 (30.0)	28 (70.0)
MTA pulpotomy	15 (37.5)	25 (62.5)	19 (47.5)	21 (52.5)
Re–root canal treatment	8 (20.0)	32 (80.0)	19 (47.5)	21 (52.5)
Fixed dental prosthesis	6 (15.0)	34 (85.0)	24 (60.0)	16 (40.0)
Prefabricated post and core	18 (45.0)	22 (55.0)	15 (37.5)	25 (62.5)
Interim removable dental prosthesis	13 (32.5)	27 (67.5)	26 (65.0)	14 (35.0)
Removable dental prosthesis	11 (27.5)	29 (72.5)	22 (55.0)	18 (45.0)

**Table 4:** Association between demographic variables of patients with their responses.

Variables		Crown Lengthening		Interim Removable Dental Prosthesis		Removable Dental Prosthesis	
		Yes *n* (%)	No *n* (%)	Yes *n* (%)	No *n* (%)	Yes *n* (%)	No *n* (%)
Gender	Female	1 (50.0)	12 (66.7)	7 (58.3)	6 (75.0)	8 (66.7)	5 (62.5)
	Male	1 (50.0)	6 (33.3)	5 (41.7)	2 (25.0)	4 (33.3)	3 (37.5)
*p*-value		0.639		0.444		0.848	
Age	18–25 years	0 (0.0)	10 (55.6)	5 (41.7)	5 (62.5)	4 (33.3)	6 (75.0)
	26–35 years	2 (100.0)	2 (11.1)	3 (25.0)	1 (12.5)	4 (33.3)	0 (0.0)
	36–45 years	0 (0.0)	6 (33.3)	4 (33.3)	2 (25.0)	4 (33.3)	2 (25.0)
*p*-value		0.012 *		0.637		0.108	
Educational level	Diploma/Bachelor’s	1 (50.0)	12 (66.7)	11 (91.7)	2 (25.0)	10 (83.3)	3 (37.5)
	High School	1 (50.0)	5 (27.8)	0 (0.0)	6 (75.0)	1 (8.3)	5 (62.5)
	Incomplete	0 (0.0)	1 (5.6)	1 (8.3)	0 (0.0)	1 (8.3)	0 (0.0)
*p*-value		0.785		0.002 *		0.032 *	

* Statistical significance set at 0.05.

**Table 5:** Association between demographic variables of students with their responses.

Variables		Crown Lengthening		Pit and Fissure Sealant		Fixed Dental Prosthesis		Prefabricated Post and Core	
		Yes *n* (%)	No *n* (%)	Yes *n* (%)	No *n* (%)	Yes *n* (%)	No *n* (%)	Yes *n* (%)	No *n* (%)
Gender	Female	19 (79.2)	5 (20.8)	11 (45.8)	13 (54.2)	6 (25.0)	18 (75.0)	11 (45.8)	13 (54.2)
	Male	10 (62.5)	6 (37.5)	6 (37.5)	10 (62.5)	0 (0.0)	16 (100.0)	7 (43.8)	9 (56.2)
*p*-value		0.247		0.519		0.030 *		0.897	
Academic year	5th year	10 (52.6)	9 (47.4)	4 (21.1)	15 (78.9)	3 (15.8)	16 (84.2)	4 (21.1)	15 (78.9)
	6th year	19 (90.5)	2 (9.5)	0 (0.0)	21 (100.0)	3 (14.3)	18 (85.7)	14 (66.7)	7 (33.3)
*p*-value		0.007 *		0.027 *		0.894		0.004 *	

* Statistical significance set at 0.05.

**Table 6:** Responses of the patients to various questions were presented under the themes.

Themes	Codes	Example	Frequency (%)
Plaque-induced gingivitis	Inflammation due to plaque/bacteria	Gingivitis due to tartar and bacteria; inflammation caused by the buildup of plaque, swelling, and bleeding of the gums	3 (33.3)
	Unspecified inflammation	Swelling, pain and redness; Inflamed and bleeding gums	6 (66.7)
Gingival recession	Gum Recession	Tooth root exposure; the pulling back of gum tissue from the teeth	13 (62.0)
	Bleeding	Blood in the gums	2 (10.0)
	Gum Expansion	Gum expansion; elevation	3 (14.0)
	Uncertain Responses	To retreat; Tight gums; Its decline; A lot of lime	4 (19.0)
Periodontitis	Inflammation and swelling	Long-term inflammation of the gum tissue; swelling; inflammation of the supportive tissues	15 (65.0)
	Discoloration and Redness	Color change; redness; discoloration	6 (26.0)
	Bleeding	Permanent bleeding; blood	9 (39.0)
	Sensitivity	Sensitivity to food and when brushing; Sensitive gums	3 (13.0)
	Pain	Pain	2 (09.0)
	Halitosis	Bad odor	1 (04.0)
Crown lengthening	Gum or bone cutting	Surgical procedure to expose tooth by removing gum tissue, and sometimes bone, to improve the appearance of the tooth or make it easier to treat; bone removal	3 (75.0)
	Restoring teeth	Restoring severely damaged teeth	1 (25.0)
GIC sandwich technique	Related to permanent restoration	Permanent method; is a technique in which a layer of glass ionomer cement is used as a base under a composite filling to provide additional protection to the tooth and enhance adhesion	3 (13.0)
	Related to temporary restoration	Temporary filling. temporary	13 (57.0)
	Not related to restoration	Deep caries that requires constant support. Tooth cleaning; Treatment is being changed	7 (30.0)
Pit and fissure sealant	Caries Preventive agent/Protective agent	A thin layer placed over the cracks and grooves of back teeth to protect them by preventing the accumulation of bacteria and food debris; to protect molars from decay; Dental protection	13 (57.0)
	Restorative agent	Used mostly for mild tooth decay, Filling. Surface filling	6 (26.0)
	Related to pulp	Nerve protection	1 (04.0)
	Fracture preventive agent	Covering cracks; tooth does not break. keeps the tooth together	3 (13.0)
Indirect pulp capping	Pulp protection	A procedure for teeth suffering from deep decay close to the nerve without exposing it. nerve protection. Indirect pulp capping is a procedure used to protect the pulp of a tooth from decay. Decay is removed and a protective material is placed avoid the need for root canal treatment.	3 (60.0)
	Other responses	Tooth nerve extraction. nerve treatment	2 (40.0)
MTA pulpotomy	Related to pulp therapy	To treat deep tooth decay that approaches the nerve without reaching it; part of the diseased nerve or pulp inside the tooth is removed, to avoid complete root canal treatment; Treatment of the nerve after removing part of it and placing a protective material and filling; nerve removal; partial	12 (63.0)
	Other responses	Cutting. Cut a part to go away the pain. Nerve inflammation, caries that extend to the nerve	7 (37.0)
Retreatment of root canal tooth	Retreatment	Procedure when initial root canal treatment fails to eliminate infection or inflammation, or when new problems appear in the treated tooth; Re-treatment of the nerve after the removal of the filling; procedure performed when previous root canal treatment fails, where the root canal is cleaned again and infected or contaminated tissue is removed, then it is filled to preserve the tooth	12 (63.0)
	Root canal treatment	Cleaning the canal, extracting the nerve and closing it. nerve treatment	4 (21.0)
	Others	Temporary filling. They bring it back again	3 (16.0)
Internal bleaching	Whitening	A substance placed on the tooth to remove the yellow outer layer; if the tooth color changes after root canal treatment; internal tooth whitening is a treatment process used to lighten the color of teeth from the inside due to injury or root canal treatment; inserting a bleaching material into the root to lighten the tooth.	8 (67.0)
	Related to RCT	After removing the abscess. After root canal treatment	2 (17.0)
	Cleaning	Cleaning	2 (17.0)
Pre-fabricated post and core	Procedure done after RCT	After root canal treatment. After root canal	3 (20.0)
	Supports tooth	Treating teeth that have lost a significant amount of their structure. A post is placed inside the tooth root to support it, and the pulp (nerve) is then treated to stabilize the tooth before a crown is placed; Strengthen the tooth; Fix the tooth so it does not break; Hold the tooth	11 (73.0)
	Other responses	Veneer support	1 (07.0)
Direct inlay/onlay restoration	Related to filling the tooth	A filling that covers the tooth of teeth part lost due to decay or a broken tooth. A restoration that is prepared and shaped directly inside the mouth using a resin or amalgam, to fill the space created by decay or fracture;	3 (38.0)
	Related to pulp	Covering of the nerve when there is direct exposure of the tooth pulp, either due to deep caries or because of an error during caries removal	2 (25.0)
	Uncertain	Ready. Types of expensive type.	3 (38.0)
Removable dental prosthesis	Temporary	Custom dental molds that can be removed and reinstalled; Dentures that the patient can put on and remove themselves; I can remove it; It is a replacement for several teeth and is removed and reinstalled by the patient; Removable prosthetics are easily removable prosthetic devices used to replace missing teeth, such as complete or partial dentures.	9 (41.0)
	Others	Animated for decoration. The dressing is falling, and we are lifted up. After removing the tooth, we make molds	13 (59.0)
Interim removable dental prosthesis	Temporary replacement	A temporary dental mold until the final dental mold is completed; Dentures are temporary replacements for missing or damaged teeth; For short time; After tooth extraction to compensate for the space, an alternative to implants; temporary alternative	20 (77.0)
	Others	For adults, Movable and placed on food; They do not have teeth	6 (23.0)
Fixed dental prosthesis	Fixed replacement	Artificial device that is permanently attached to the mouth to replace missing teeth, such as a bridge or crown, and can only be removed by a dentist; Dentures are permanently attached to replace missing or damaged teeth; compensatory bridge between years after completing their treatment and compensating for the missing between them. It is done in lab	6 (25.0)
	Temporary replacement	Fixed dressing. Fixed for temporary tooth	2 (08.0)
	Other responses	Combinations; Crown; Post root canal treatment formula; Protects teeth; Full tooth veneer	16 (67.0)

## Data Availability

All the data of this research are included and presented in the article.
